# A linear purpuric rash in an elderly man

**DOI:** 10.1016/j.jdcr.2025.03.022

**Published:** 2025-04-08

**Authors:** Vinh Nguyen, Christopher N. Nguyen, Dario Kivelevitch

**Affiliations:** a School of Medicine, Texas A&M College of Medicine, Dallas, Texas; b Department of Dermatology, Baylor University Medical Center, Dallas, Texas

**Keywords:** blaschkoid, lichenoid pigmented purpuric dermatosis, linear, pigmented purpuric dermatosis, PPD

## Case presentation

**Fig 1:**
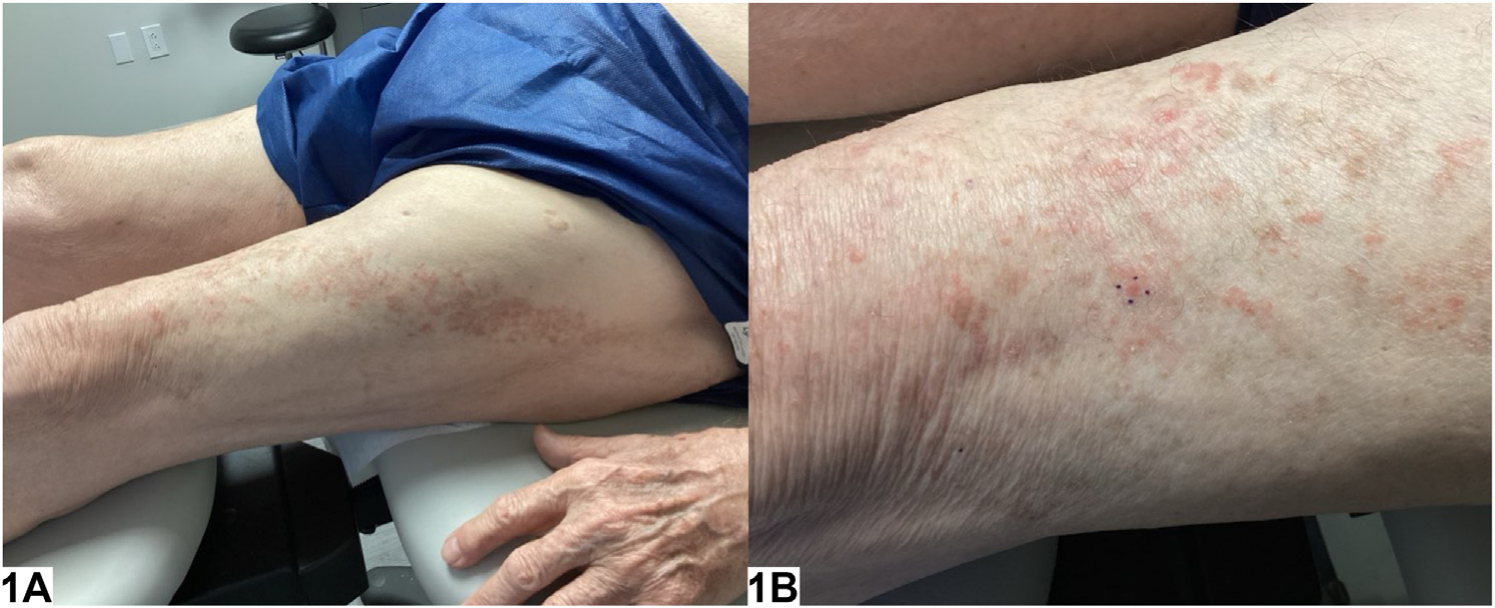

**Fig 2:**
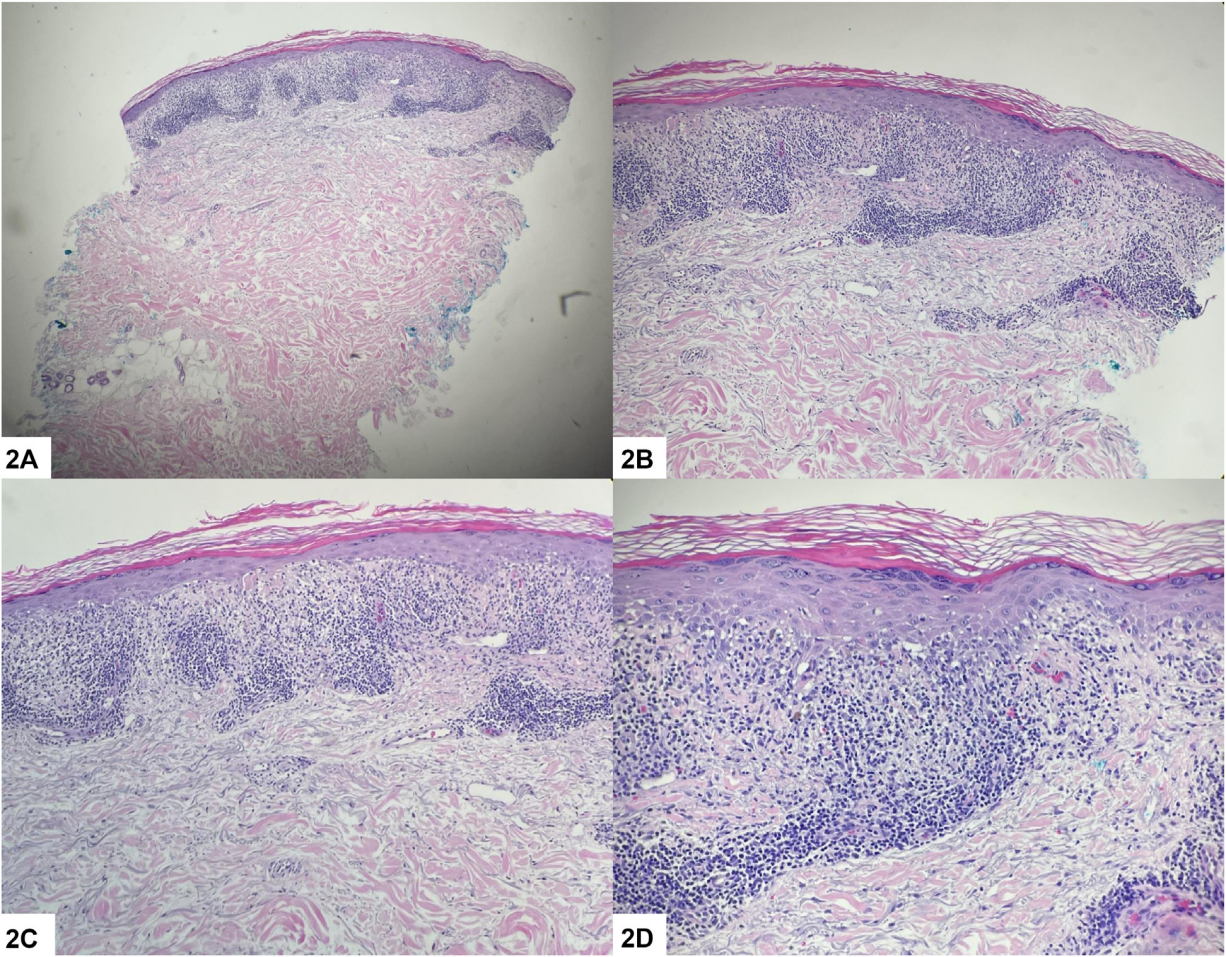

An 88-year-old male presented to the dermatology clinic for evaluation of a rash on his left thigh. This rash had been present for several months and was asymptomatic. His primary care physician diagnosed the rash as atopic dermatitis; however, there was no improvement with emollients or triamcinolone 0.1% cream. He denied a history of herpes zoster and had received the shingles vaccine. On physical exam, there were red-brown papules coalescing into plaques in a linear distribution across his left thigh (Fig 1). A punch biopsy showed band-like lymphocytic infiltrate at the dermal-epidermal junction, along with erythrocyte extravasation and hemosiderin deposition (Fig 2).

**Question 1: what is the diagnosis?**

A. Linear lichenoid pigmented purpura
B. Lichen striatus
C. Linear lichen planus
D. Linear Darier disease
E. Linear psoriasis

**Answers:**

A. Linear lichenoid pigmented purpura – Correct. Pigmented purpuric dermatosis (PPD) describes a group of chronic skin disorders characterized by distinct red-to-golden brown macules, patches, and papules with petechiae.^1^^,^^2^ PPD is generally divided into 55 major variants, with linear pigmented purpura – also known as unilateral linear capillaritis – being a rarer form. It often presents unilaterally on an extremity, sometimes in a blaschkoid distribution such as in our patient.^1^^,^^3^^,^^4^ On histology, lymphocytic inflammation with extravasated erythrocytes in the upper dermis is seen.^1^^,^^4^ Our patient’s histology was consistent with a lichenoid pattern, helping further classify the diagnosis as the even more rare linear lichenoid pigmented purpura.^4^
B. Lichen striatus – Incorrect. Lichen striatus primarily occurs in children, and skin biopsy shows peri-eccrine lymphocytic inflammation.^4^
C. Linear lichen planus – Incorrect. Linear lichen planus presents with significant pruritus, deep purple-colored lesions, and postinflammatory hyperpigmentation.^4^ In addition, histology shows a lichenoid band of inflammation in the papillary dermis with epidermal hyperplasia and hypergranulosis but without extravasated erythrocytes.^4^
D. Linear Darier disease – Incorrect. Segmental Darier disease is typically composed of keratotic, sometimes crusted, papules that are pink to red-brown rather than purpuric. Histopathologic findings include acantholysis and dyskeratosis.
E. Linear psoriasis – Incorrect. Linear psoriasis typically presents as intensely pruritic, erythematous, and scaly papules and plaques along Blaschko’s lines. Biopsy would not show a lichenoid infiltrate.^5^

**Question 2: which is the most common underlying cause of this condition?**

A. Hepatitis C
B. Medication-induced
C. Liver disease
D. Genetic mosaicism
E. Idiopathic

**Answers:**

A. Hepatitis C – Incorrect. Hepatitis C is associated with the development of lichen planus.
B. Medication-induced – Incorrect. Although medications are frequently reported as triggers for PPD, the etiology of PPD is unknown.^1^ Identified medication triggers include acetaminophen, aspirin, chlordiazepoxide, glipizide, and hydralazine.^1^^,^^2^^,^^4^
C. Liver disease – Incorrect. PPD is rarely associated with systemic disorders such as hyperlipidemia, diabetes mellitus, rheumatoid arthritis, thyroid disease, liver disease, and hematologic or solid neoplasms.^2^
D. Genetic mosaicism – Incorrect. Genetic mosaicism is believed to play a role in the pathogenesis of linear psoriasis, although the exact mechanism is not completely understood.^5^
E. Idiopathic – Correct. Most cases of PPD are idiopathic. PPD results from minimal inflammation and hemorrhage of vessels of the superficial dermis, most commonly capillaries, in the absence of coagulopathy.^2^^,^^3^ Physical activity, prolonged standing, venous hypertension, capillary fragility, and local infections are underlying factors that may lead to the development or worsen preexisting PPD.^2^

**Question 3: what other variants of this entity can also present with lichenoid inflammation on histopathology?**

A. Lichen aureus
B. Pigmented purpuric lichenoid dermatitis of Gougerot and Blum
C. Purpura annularis telangiectodes of Majocchi
D. A & B
E. A, B, & C

**Answers:**

A. Lichen aureus – Incorrect. Although lichen aureus does have a lichenoid infiltrate, this is not the only variant of PPD that may show the lichenoid pattern.^2^^,^^4^
B. Pigmented purpuric lichenoid dermatitis of Gougerot and Blum – Incorrect. Similar to lichen aureus, pigmented purpuric lichenoid dermatitis of Gougerot and Blum may also have a lichenoid infiltrate.^2^^,^^4^
C. Purpura annularis telangiectodes of Majocchi – Incorrect. Purpura annularis telangiectodes of Majocchi does not typically have a lichenoid infiltrate, but rather a perivascular pattern of lymphocytic inflammation, which is similar to Schamberg disease.^2^
D. A & B – Correct. Both of those variants exhibit a lichenoid inflammatory pattern.^3^
E. A, B, & C – Incorrect. See explanation for answer choice C.^3^

## Conflicts of interest

None disclosed.
